# Making fundamental scientific discoveries by combining information from literature, databases, and computational tools – An example

**DOI:** 10.1016/j.csbj.2021.04.052

**Published:** 2021-05-14

**Authors:** Bastian Stielow, Clara Simon, Robert Liefke

**Affiliations:** a Institute of Molecular Biology and Tumor Research (IMT), Philipps University of Marburg, 35043 Marburg, Germany; b Department of Hematology, Oncology and Immunology, University Hospital Giessen and Marburg, 35043 Marburg, Germany

**Keywords:** Databases, Protein structure prediction, SAMD1, CpG islands, Transcription factors, Human genome

## Abstract

In recent years, the amount of available literature, data and computational tools has increased exponentially, providing opportunities and challenges to make use of this vast amount of material. Here, we describe how we utilized publicly available information to identify the previously hardly characterized protein SAMD1 (SAM domain-containing protein 1) as a novel unmethylated CpG island-binding protein. This discovery is an example, how the richness of material and tools on the internet can be used to make scientific breakthroughs, but also the hurdles that may occur. Specifically, we discuss how the misrepresentation of SAMD1 in literature and databases may have prevented an earlier characterization of this protein and we address what can be learned from this example.

## 1 Introduction

**Fig. 1:**
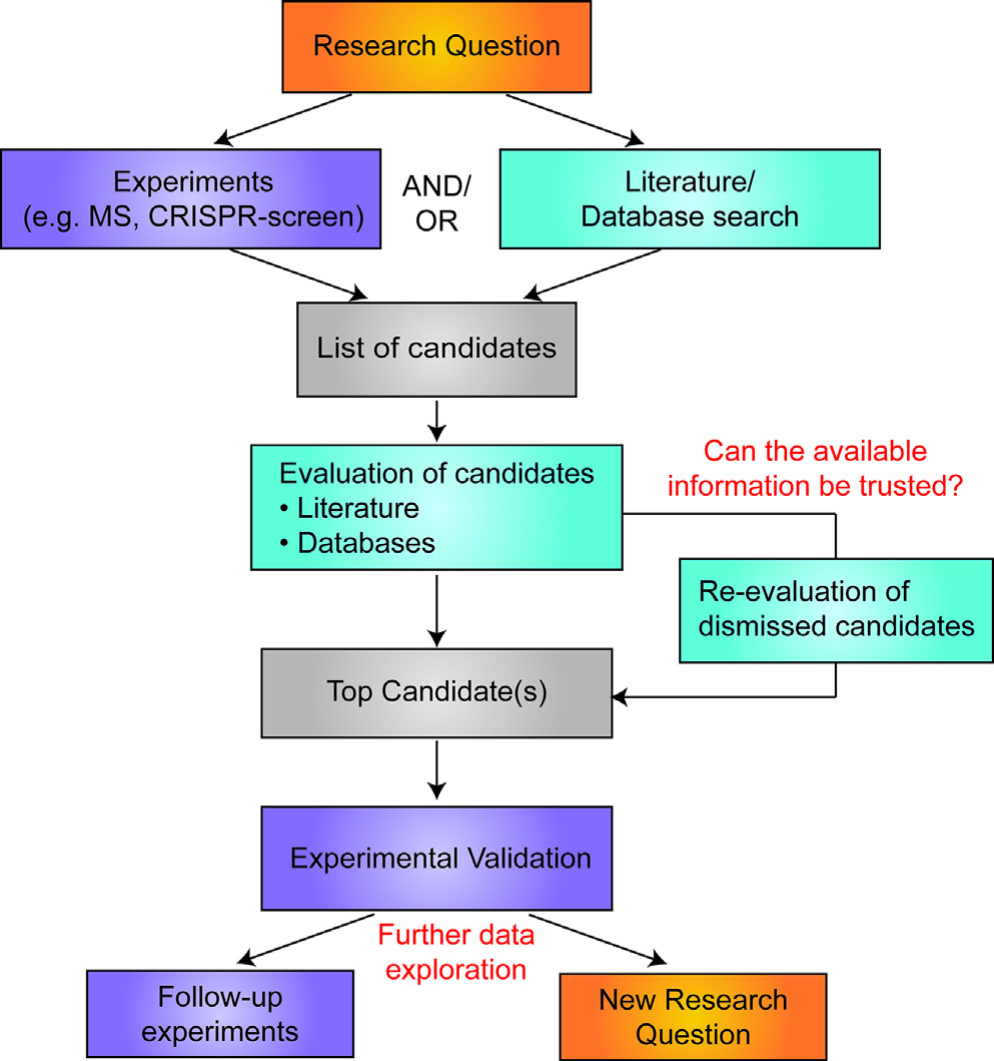
Outline how a scientific question can be addressed. Besides doing experiments, the investigation of literature and accessible databases may allow to identify suitable candidates for follow up investigations. Information in databases is not always reliable and requires careful evaluation. Closer inspection of dismissed candidates may reveal suitable candidates for an in-depth investigation. After further validation, utilizing databases and computational tools may provide the ground for follow-up experiments or to develop novel research questions.

The function of a cell is mainly dictated by proteins and their interplay. Therefore, the characterization of protein functions during various biological processes is one of the fundamental research tasks in life science. To learn more about proteins and to discover potential novel functions, several alternative strategies can be applied. Often, novel findings build on screening experiments, which are designed to fish out proteins or genes that may play a role in a certain context. Subsequently, the potential candidates are investigated whether they indeed have a function relevant to the original research question. However, novel discoveries may not always rely on experimental approaches. The enormous increase of literature and data provides the ground to discover interesting proteins or protein functions solely by the correct interpretation of these accessible material. Thus, instead of uncovering candidates by performing specific experiments, it becomes more and more feasible to search for potential candidates by investigating publicly available material (Fig. 1).

Here, we present as an example, how the usage of public material led to the discovery of a novel CpG island-binding protein, SAMD1 (SAM domain-containing protein 1). This protein has hardly been characterized yet, and the information in databases were partially incomplete or wrong (stand May 2021). This wrong information may have contributed to the hesitance of researchers to investigate this protein in more detail. Nonetheless, despite not much was known about SAMD1, the combination of numerous hints led to the conclusion that SAMD1 is a strong candidate to be a novel unmethylated CpG island-binding protein. Based on this data exploration, we could indeed subsequently confirm our hypothesis using extensive structural, molecular biological and genome-wide approaches [1]. In this article, we describe how we detected SAMD1 in the first place, and how the exploration of publicly available material and the usage of bioinformatic tools increased our confidence that SAMD1 is a worthy candidate for in-depth investigation.

### 1.1 Defining a research question

To address a specific question, it is essential either to design appropriate experiments, or to survey the literature and/or public data towards a specific aspect (Fig. 1). The here discussed research question emerged from the structural work of the Zhanxin Wang group, who identified the Polycomb-like proteins as novel proteins that specifically can bind to unmethylated CpG-motifs [2]. The CpG -motif is often enriched at so-called CpG islands (CGIs), which are commonly found at promoters and play important gene regulatory roles [3]. CGIs are either in a methylated state, which leads to transcriptional silencing, or in an unmethylated state, where the respective gene is typically actively transcribed. Before the discovery of the Polycomb-like proteins, only the CXXC-domains have been described to specifically interact with unmethylated, but not with methylated CpG-motifs [4], [5]. Proteins containing CXXC-domains are often part of larger protein complexes that can modulate the chromatin status. For example, CXXC1 (CXXC Finger Protein 1; also CFP1) is a component of the COMPASS (Complex Proteins Associated with Set1) complex [6], which is important to establish the active H3K4me3 mark at promoters. The discovery that besides the CXXC-domain proteins also the Polycomb-like proteins can bind to unmethylated CpG-motifs, offered several novel aspects about the regulatory function of the CGIs. First, this work provided an explanation for the known localization of the Polycomb repressive complex 2 (PRC2) at CGIs [7], [8]. Second, it emphasized that unmethylated CGIs can be involved both in gene activation and gene repression [2]. Thirdly, this work showed that besides CXXC-domains also other domains can bind to unmethylated CpG-motifs. The Polycomb-like proteins facilitate the CpG-binding function via a winged-helix domain. Intriguingly, the overall fold of the CXXC-domains and the winged-helix domain is fundamentally different, yet the CpG recognition is remarkably similar, mainly established by a loop of charged amino acids that reach into the major groove of the DNA [2]. This finding suggests that the CpG-motif recognition is more flexible than previously assumed, and that a CpG-binding loop can possibly be created also by other domains. Thus, the question arose whether other so far undescribed proteins or domains may facilitate the interaction with unmethylated CpG-motifs.

### 1.2 Finding an unexpected protein

**Fig. 2:**
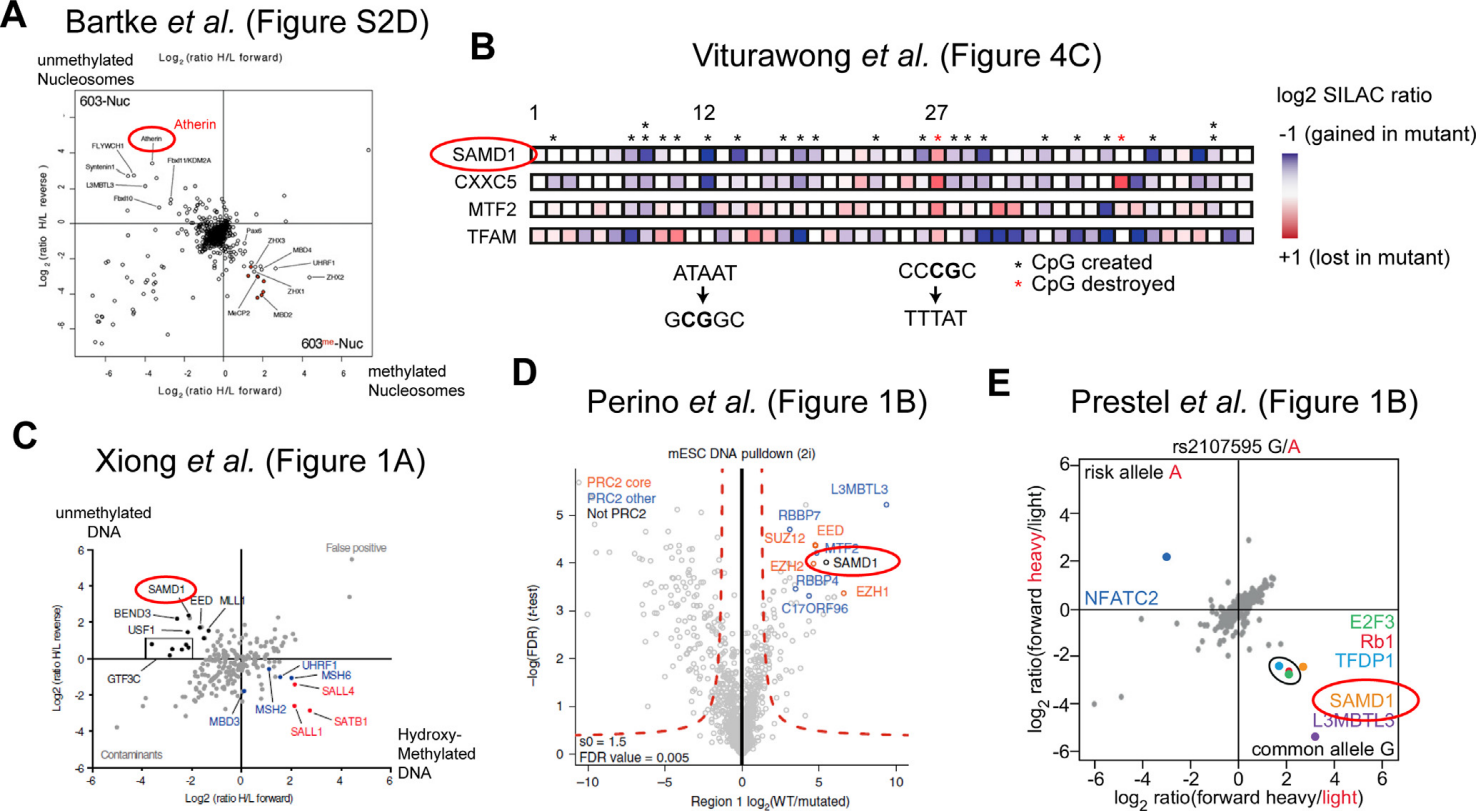
A-E) DNA-related occurrences of SAMD1 in the literature [9], [13], [14], [15], [16]. Those publications cannot be found by standard literature search but were discovered via internet research. In B) only selected parts of the figure are shown and for each column, the creation or disappearance of a CpG-motif upon transitional DNA mutations, is presented by asterisks. For columns 12 and 27 the DNA change is presented below. Together, these publications suggest a preference of SAMD1 for CpG-rich and unmethylated DNA.

As already numerous studies, which have explored chromatin- and DNA-binding proteins, are avaible we wondered whether these data are sufficient to identify unmethylated CpG-motifs candidates. Thus, instead of establishing and performing an experiment to identify novel CpG-binding proteins, we decided to investigate first the available material for putative candidates (Fig. 1). Which criteria may be suitable to identify novel CpG-binding proteins? We assumed that such proteins should be localized nuclear, associated with chromatin and they should preferentially bind to unmethylated and CpG-rich DNA. How can such factors be identified? The easiest way appears to search for publications, in which proteins are investigated for their preference either for methylated or for unmethylated DNA. One of the earliest publications in this regard was Bartke *et al.* [9], which explored nucleosome-binding proteins dependent on both, DNA methylation, and histone modification status using mass-spectrometry. Surveying the accompanying tables and supplementary figures from this study, we were looking for proteins that are excluded from nucleosomes with methylated DNA. Besides known unmethylated CpG-binding proteins, such as MTF2 (Metal Response Element Binding Transcription Factor 2), FBXL10 (F-Box and Leucine-Rich Repeat Protein 10; also KDM2B), and CXXC5 (CXXC Finger Protein 5), several other proteins are associated specifically with unmethylated nucleosomes. Unexpectedly, those proteins were not only known chromatin-associated proteins, but also other less known proteins. In particular, a protein called “Atherin” (also named SAMD1) drew our attention, because it belongs to the proteins, which are most strongly excluded from methylated nucleosomes (Fig. 2A). The name “Atherin” refers to the first description of this protein, as a secreted protein involved in atherosclerosis [10]. Besides this one publication dated almost 20 years ago, no further evidence supports a role of Atherin in atherosclerosis. The occurrence of Atherin in mass-spectrometry datasets of nucleosome-associated proteins either suggests that it is a contamination or that Atherin may have a completely distinct function than originally described. Given that Atherin is not highly enriched in the CRAPome database [11], which identifies typical contaminants from mass-spectrometry experiments, we speculated that the second possibility could be true. For this reason, we will use the more neutral term “SAMD1” (SAM domain-containing protein 1) for further description of this protein.

### 1.3 The pitfalls of using internet search engines

To further investigate SAMD1’s potential role at DNA and/or chromatin, we looked for additional publications where SAMD1 is occurring. However, as SAMD1 has hardly been characterized yet, not many publications about SAMD1 are available in the literature databases. The only publications about SAMD1 refer to the SAM-domain function [12], but the authors did not address the role of SAMD1 specifically. Similarly, searching for SAMD1 using internet search engines mainly leads to hits from databases, such as UniProt (universal protein database) or NCBI (National Center for Biotechnology Information), but hardly to any publications. In addition, we recognized that the close similarity of the name to the well-characterized protein SMAD1 (Suppressor of Mothers against Decapentaplegic 1) complicates the search for SAMD1-containing literature, since many hits refer to SMAD1, instead of SAMD1. Also, in not a few number of publications “SMAD1” is misspelled as “SAMD1” leading to false-positive hits. Nonetheless, we found that excluding SMAD1 using “-SMAD1” as additional search criteria, strongly improved the internet search results. Given that SAMD1 has hardly been investigated yet, we speculated that SAMD1 may predominantly occur in large-scale datasets and is perhaps hidden in supplementary data or figures. Since we are mainly interested in the SAMD1 protein, and not its transcript, we searched for protein-related datasets. Indeed, using the search “SAMD1 -SMAD1 mass-spectrometry” led to several interesting findings.

### 1.4 More evidence that SAMD1 could be a novel CpG-binding protein

One of the found publication was Viturawong *et al.* [13], which investigated proteins that bind to ultra-conserved genomic regions. In one figure where the authors systematically analyzed the consequence of transitional DNA changes (A<>G, C<>T) on the DNA-bound proteins via mass-spectrometry, SAMD1 levels were found to be altered upon several DNA changes. To comprehend which transformation of the DNA sequence led to changed SAMD1-binding, we systematically assessed the alteration of the DNA sequence (Fig. 2B). Remarkably, we found that an increase of SAMD1-binding often correlates with the occurrence of novel CpG-motifs (position 12), while a decrease is associated with the disappearance of a CpG-motif (position 27). This pattern is similar to MTF2, one of the Polycomb-like proteins, and CXXC5, a CXXC-domain-containing protein. Other proteins, such as TFAM, show totally different patterns. Further search identified more publications where SAMD1 is found in mass-spectrometry. In Xiong *et al.* [14], SAMD1 was repelled from hydroxy-methylated DNA (Fig. 2C), while in Perino *et al.* [15] SAMD1 was pulled-down by CpG-rich DNA (Fig. 2D). Further, SAMD1 has also been found to be affected by mutations of CpG-containing E2F binding motifs [16]. Here, a mutation of a CpG-motif within the E2F binding sites (TTTCC**CG**CA to TTTCC**CA**CA), led to strong reduction of SAMD1 DNA-binding (Fig. 2E). Notably, in many of those publications, SAMD1 and the protein L3MBTL3 behaved rather similar, suggesting that SAMD1 and L3MBTL3 (Lethal(3)malignant brain tumor-like protein 3) are connected. Indeed, in pull-down experiments using SFMBT1 as bait, SAMD1 and L3MBTL3 were co-eluted [17], suggesting that they are both parts of a larger protein complex. These data also demonstrate that SAMD1 is likely associated with chromatin-related protein complexes, supporting our original idea.

Altogether, the survey for publications provided several independent evidences that SAMD1 has a preference to interact with unmethylated CpG-motifs (Fig. 2), making it a potential candidate for our original research question.

### 1.5 How to deal with conflicting gene annotations?

Due to these first hints suggesting that SAMD1 could be a novel CpG-motif binding protein, we aimed to learn more about SAMD1**.** However, when we attempted to gather further information about SAMD1, we recognized some problems regarding the annotation of the SAMD1 gene. The following information is provided by UniProt (ID: Q6SPF0) [18] about the human SAMD1 gene: “Due to its high GC-content it turned out to be difficult to sequence the 5′ end of the gene encoding the N-terminal proline-rich region of the protein and to unambiguously determine which sequence is correct. We display the sequence described in PubMed: 16159594. This sequence fits better with orthologous sequences but is not consistent with the human reference genome sequence”. So, we had a closer look at the DNA sequence provided by PubMed:16159594 [10], which has been uploaded to NCBI (ID: 38565228). Looking at the sequences, one can easily recognize that the cDNA sequence of SAMD1 is indeed extremely CG-rich, suggesting that sequencing of the SAMD1 gene is not trivial. Consequently, automated genome assembly may have mis-annotated SAMD1, which was then introduced into the databases. Comparing the database entry of human SAMD1 in UniProt and Ensembl (ID: ENSG00000141858) [19] demonstrates the problem. In UniProt, human SAMD1 has a length of 538 amino acids, while in Ensembl it is only 432 amino acids. UniProt refers to the sequence originally described by Lees *et al.* [10], while Ensembl is based on the automated genome assembly. Interestingly, more recent investigations on the human genome using modern technology, such as SMRT-Seq [20], identified this discrepancy. In several publications [21], [22], [23] a 318 bp insertion to the SAMD1 gene is mentioned, which reflects exactly the 106 amino acids difference between the two annotations in UniProt and Ensembl. Thus, we concluded that the 518 amino acids sequence for human SAMD1, as originally described in Lees *et al.* [10], is likely the correct sequence.

### 1.6 There is no DNA-binding domain in SAMD1. Really?

**Fig. 3:**
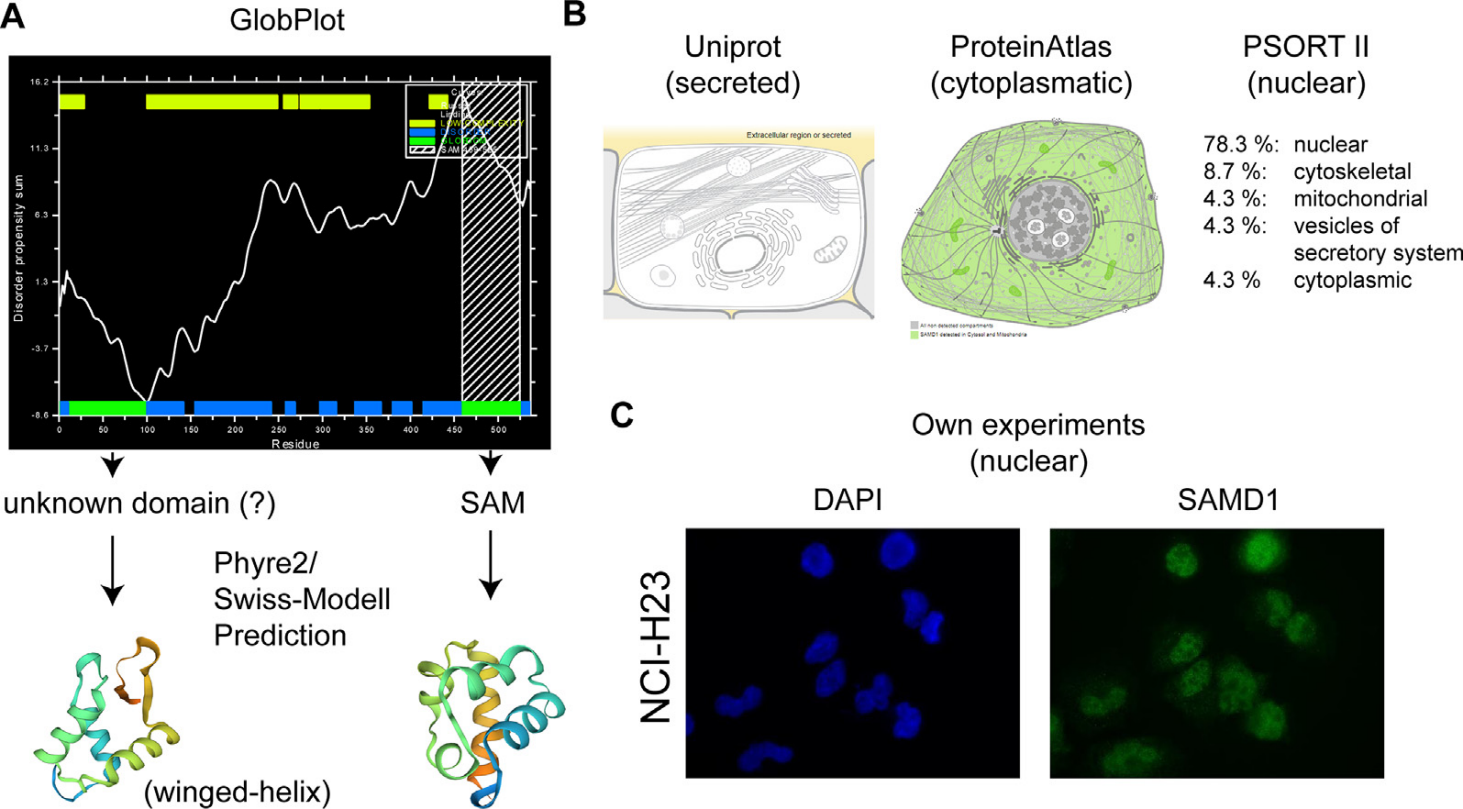
A) Discovery of an additional globular domain at the N-terminus of SAMD1 using GlobPlot [26]. Follow-up investigations of the N-terminal domain using structure prediction software, such as Phyre2 [27] and SWISS-MODEL [28], identifying this domain as a putative winged-helix domain. B) Discrepancy about the cellular localization of SAMD1 between UniProt [18], Protein Atlas [29], and the PSORT II prediction tool [30]. C) Own experiments strongly support a nuclear localization of SAMD1. See also Stielow *et al.*[1].

Having decided which annotation for SAMD1 we should use for further investigations, it was now important to nail down whether SAMD1 indeed can bind to CpG-rich DNA. Specifically, we asked whether SAMD1 possesses a domain that is suitable for DNA binding. So far only one domain is annotated in SAMD1, namely the C-terminal SAM-domain. SAM-domains are commonly involved in protein–protein interactions and are not typically participating in DNA binding [24], implying the SAM-domain cannot contribute to the potential binding of SAMD1 to CpG-rich sequences. SMART (Simple Modular Architecture Research Tool) [25] and comparable tools showed no other domains in SAMD1, suggesting that the rest of the protein has no region that is similar to known domains. Interestingly, however, the GlobPlot tool [26] predicted an additional globular domain at the N-terminus of SAMD1 (Fig. 3A), suggesting that SAMD1 indeed has another domain, besides its C-terminal SAM-domain. But what kind of domain is it? To address this question, we made use of protein structure prediction software. Remarkably, Phyre2 [27] and SWISS-MODEL [28] predicted with high confidence that the N-terminal globular domain is a winged-helix domain, most similar to histone H1 (Fig. 3A). Interestingly, the primary sequence shows only a very low sequence identity to other winged-helix domains, possibly explaining why this domain has not been identified by other domain prediction software. This example therefore demonstrates that the usage of protein structure prediction software may reveal unannotated domains, that have distinct primary sequences, but a similar fold to known domains.

Thus, this investigation led to the discovery of a putative novel winged-helix domain in SAMD1, which has not been annotated before. Given that the Polycomb-like proteins bind to unmethylated CpG-motifs also via a winged-helix domain [2], we speculated that the winged-helix domain of SAMD1 can possibly facilitate interaction to CpG-rich DNA, as well.

### 1.7 Secreted, cytoplasmatic, nuclear?

As mentioned before, SAMD1 has been described to be a secreted protein [10]. This finding has also been imported into UniProt and other databases (Fig. 3B). In contrast, the Protein Atlas [29], a comprehensive database about proteins, describes the cellular localization of SAMD1 entirely differently. Here, SAMD1 is presented as a cytoplasmatic protein, supported by a strong cytoplasmatic staining using custom-made antibodies (Fig. 3B). On the other hand, the PSORT II software [30] predicts SAMD1 to be nuclear with 78.3 percent confidence. Also, the presence of SAMD1 in several chromatin related publications [17], [31], [32] supports a nuclear localization of SAMD1. So, where is SAMD1 really localized? Given this discrepancy between the information, we decided ultimately to address this issue experimentally. We created our own SAMD1 antibody [1] and by using immunofluorescence, we found that in all investigated cells, SAMD1 is mostly present in the nucleus (Fig. 3C), confirming the PSORT II prediction. This result disagrees with the Protein Atlas and with the description of SAMD1 to be secreted [10]. How can these contradicting findings be explained? Most likely, the used antibodies may not be fully specific for SAMD1 and therefore the obtained signal reflects something SAMD1-unrelated. Notably, Protein Atlas stainings of SAMD1 in human tissues, which were made with the same antibody as for the cell lines, clearly shows a nuclear localization of SAMD1. Why the same antibody leads to totally different staining in cell lines and tissues has not yet been resolved by the Protein Atlas database.

### 1.8 From research question, to candidate, to experimental validation

In summary, starting from the question of whether there are more CpG-binding proteins, we explored literature and came across SAMD1 as a potential candidate. It was an unexpected finding, given that SAMD1 has been described to be secreted and to be involved in Atherosclerosis [10]. However, upon closer inspection, we could gather further evidence that this protein is linked to unmethylated CpG-rich DNA (Fig. 2). We also detected a putative winged-helix domain (Fig. 3A) and found that SAMD1 is mainly nuclear (Fig. 3C). Interestingly, none of this information can be found easily, since the data about SAMD1 are either deeply buried in publications, incomplete or wrong. However, our survey led to the conclusion that SAMD1 is a strong candidate for further investigations. Ultimately, we were indeed able to confirm a chromatin regulatory role of SAMD1 at unmethylated CGIs [1]. Together with the group of Zhanxin Wang we could solve the structure of SAMD1′s winged-helix domain with CpG-containing DNA (PDB: 6LUI) and we could demonstrate that SAMD1 binds to a subset of unmethylated CGIs in mouse embryonic stem cells, where it carries out a repressive function [1]. Thus, our work could demonstrate that beyond the CXXC-domain proteins and the Polycomb-like proteins indeed further proteins are able to bind unmethylated CGIs, which confirmed our original research question. Notably, SAMD1 is a unique chromatin regulator, because it not only binds to CGIs, but it is also connected to SAM-domain proteins, which play a role in long-range chromatin interactions [33], [34]. This combination may allow SAMD1 to facilitate a special function at CGIs. Thus, our discovery will help to better understand CpG island regulation in the future.

### 1.9 What else can be learned from internet databases?

Despite our structural and molecular biological characterization of SAMD1, many aspects of SAMD1 remain to be explored. What else can be learned about hardly characterized proteins, using the available resources? It is always useful to search for publications, where the gene/protein of interest is mentioned. Many of those publications cannot be found by standard literature search, but instead can only be detected by smart and extensive internet research, given that the relevant information is buried in the figures or the supplementary data. For SAMD1 several publications can be found, where it has been linked to certain diseases. For example, a non-synonymous nonvariant of SAMD1 has been associated via GWAS with the severity of symptoms upon a malaria infection [35]. Thus, it could be interesting to work out what are the consequences of this amino acid alteration for the biological function of SAMD1, and how this influences the response to malaria infection.

**Fig. 4:**
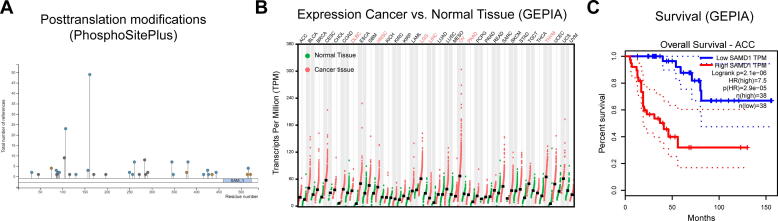
A) Posttranslational modifications (PTMs) of SAMD1, obtained by PhosphositePlus [36] B) Expression of SAMD1 in human normal and cancer tissues, based on TCGA (The Cancer Genome Atlas) data [43] and visualized by GEPIA [38]. C) Kaplan-Meier-Survival curve of ACC patients separated based on SAMD1 expression in the tumor samples. Curve obtained from GEPIA [38].

Besides looking for specific publications, we recommend making use of the many available databases in the internet to gather information about known or unknown proteins. Basic information about a protein, such as its sequence and annotated domains, are comprehensively included into the UniProt database (www.uniprot.org) [18]. An overview of identified posttranslational modifications (PTMs), such as phosphorylation, methylation, ubiquitylation and sumoylation can be found with PhosphositePlus (https://www.phosphosite.org/) [36]. For SAMD1 several such modifications have been identified (Fig. 4A), which may be important to fine-tune the function of SAMD1. The expression of a gene in human tissues can most easily been assessed using the GTEx (Genotype-Tissue Expression) database (https://gtexportal.org/) [37] or the Protein Atlas (https://www.proteinatlas.org/) [29]. SAMD1 is expressed in all human tissues (not shown), suggesting a ubiquitous function. The expression of a protein in cancer versus normal tissue can be deduced using the GEPIA platform (http://gepia.cancer-pku.cn/) [38]. An increased expression of SAMD1 in several cancer tissues can be found (Fig. 4B), which may suggest a role of SAMD1 in cancer. The relationship between gene expression and cancer patient survival can be investigated via GEPIA or the Xena Browser (https://xenabrowser.net/) [39]. For example, SAMD1 expression has a strong prognostic value for ACC (adenoid cystic carcinoma) patients (Fig. 4C), further supporting a role of SAMD1 in cancer. Cancer related point mutations are systematically presented in the cBioPortal (https://www.cbioportal.org/) [40], which is useful to identify potentially important regions for the protein function in cancer. Further, the results of CRISPR screen experiments are now included into various databases. For example, the role of proteins for the proliferation of cancer cell lines can be evaluated using DepMAP (https://depmap.org/) [41]. Results from more CRISPR-Screens can easily be accessed using BioGRID ORCS (https://orcs.thebiogrid.org/) [42]. This collection is not exhaustive, and many more databases are available that address specific scientific queries. Information from those databases may allow performing follow-up experiments to investigate further details about the protein of interest or to develop novel research questions (Fig. 1).

## 2 Conclusion

The example in this article demonstrates the strength and the pitfalls that come with the accessibility and the spread of research data. On one side, it shows the disadvantage of the automatic transfer from large-scale research data into databases, without proper curation. Due to technical challenges, such as the high CG content in the SAMD1 gene, wrong data may get introduced into databases. Those data are also often exchanged from one database to another, leading to an amplification of potentially incorrect information. For example, the (likely) erroneous annotation of SAMD1 with a protein length of 432 amino acids, which has been obtained during early human genome assemblies [44], is present in several databases, such as Ensembl and the UCSC browser. Currently in remains unclear when the SAMD1 gene will be corrected in the human genome databases and when downstream databases will reprocess their data to update their entries. This will also be relevant for other wrongly annotated genes. In addition, the questionable description of the SAMD1 protein as a secreted protein can also be found in miscellaneous databases. Thus, contradicting, or wrong information from various databases are not easy to recognize and can prevent to investigate potentially interesting proteins. This issue is particularly relevant for researchers that not extensively make use of these databases. The constant occurrence of novel databases, reorganization of established databases, and disappearance of other databases, makes it often difficult to know which databases are still up-to-date and can be trusted.

Besides those general database problems, the naming of SAMD1 as “Atherin” may have led to a misjudging of the protein. “Atherin” sounds like a typical mass-spectrometry contaminant, such as Tubulin or Albumin. SAMD1 may have been dismissed by others as a protein of interest, simply because of its name. Only a more careful investigation can reveal that SAMD1 is much less commonly found in mass-spectrometry experiments than classical contaminants, such as TUBB3 (Tubulin) and ALB (Albumin) [11]. We hope that in the future the neutral name SAMD1 will exclusively been used.

Nonetheless, despite those obstacles, the discovery of SAMD1 is a great example for the opportunities that lay in the enormous amount of freely accessible material. Starting from the initial sighting of SAMD1 in Bartke *et al.* [9], we were able to identify several additional publications that have mentioned SAMD1, simply by searching the internet. It would have been impossible to find those publications using classic literature search in the library. Also, the presence of SAMD1 in numerous databases provided the ground to investigate this protein in more detail. It allowed to critical assess the available information and to resolve some discrepancies, such as the gene annotation and the cellular localization. Furthermore, the discovery of the winged-helix domain was only possible because of the availability of the protein sequence and structure prediction software [27], [28], which substantially improved our confidence in SAMD1. Without those tools, SAMD1 would not have become a candidate for our research question, which probably would have delayed the characterization of SAMD1 as a novel CGI binding protein [1] for a couple of more years.

In summary, our excursion into the discovery of SAMD1 demonstrates that it is not always required to perform experiments to identify potential interesting candidates for a research question. It is possible to find exciting proteins by critical investigating the available literature, databases and by utilizing appropriate tools. Experience with those utilities will permit to exclude unsuitable candidates and to identify interesting candidates, which could allow to skip time-consuming and cost-intensive screening experiments and start directly with validation experiments, as we have done it for SAMD1 [1]. Given that this strategy can possibly accelerate the progress in science, it is advisable to be familiar with common internet databases, and computational tools, to make full use of their potential.

## CRediT authorship contribution statement

**Bastian Stielow:** Investigation, Writing - review & editing. **Clara Simon:** Investigation, Writing - review & editing. **Robert Liefke:** Conceptualization, Investigation, Writing - original draft, Visualization, Supervision, Project administration, Funding acquisition.

## Declaration of Competing Interest

The authors declare no conflict of interest.
